# Abnormalities in spontaneous abortions detected by G-banding and chromosomal microarray analysis (CMA) at a national reference laboratory

**DOI:** 10.1186/1755-8166-7-33

**Published:** 2014-05-22

**Authors:** Boris T Wang, Thomas P Chong, Fatih Z Boyar, Kimberly A Kopita, Leslie P Ross, Mohamed M El-Naggar, Trilochan Sahoo, Jia-Chi Wang, Morteza Hemmat, Mary H Haddadin, Renius Owen, Arturo L Anguiano

**Affiliations:** 1 Cytogenetics Department, Quest Diagnostics Nichols Institute, San Juan Capistrano, CA 92690, California

**Keywords:** POC, Maternal deciduous tissue, Placental villi, G-banding, CMA, UPD

## Abstract

**Background:**

Cytogenetic evaluation of products of conception (POC) for chromosomal abnormalities is central to determining the cause of pregnancy loss. We compared the test success rates in various specimen types and the frequencies of chromosomal abnormalities detected by G-banding analysis with those found by Oligo-SNP chromosomal microarray analysis (CMA). We evaluated the benefit of CMA testing in cases of failed culture growth.

**Methods:**

Conventional cytogenetic results of 5457 consecutive POC specimens were reviewed and categorized as placental villi, fetal parts, and unspecified POC tissue. The CMA was performed on 268 cases. Of those, 32 cases had concurrent G-banding results. The remaining 236 cases included 107 cases with culture failure and 129 cases evaluated by CMA alone.

**Results:**

The overall POC culture success rate was 75%, with the lowest for fetal parts (37.4%) and the highest for placental villi (81%). The abnormality rate was 58% for placental villi, but only 25% for fetal parts. Of the abnormalities detected, the most common were aneuploidies, including trisomy 16, triploidy, monosomy X, trisomy 22, trisomy 21 and trisomy 15, while the least encountered aneuploidies were trisomy 1, trisomy 19 and monosomies (except monosomy 21). Overall, POC specimens studied by CMA were successful in 89.6% of cases and yielded a 44.6% abnormality rate.

**Conclusions:**

Placental villi yielded higher rates of culture success and a higher percentage of abnormal karyotypes than did other specimen types. The Oligo-SNP CMA method has demonstrated a viable alternative to the G-banding method in view of its advantages in detection of submicroscopic genomic aberrations, shorter turnaround time due to elimination of time required for culture and a higher test success rate.

## Background

Spontaneous pregnancy loss is a common clinical occurrence. Many studies have demonstrated that 50% of all fertilized eggs die and spontaneously abort, usually before the pregnancy is recognized. Among women who know they are pregnant, the miscarriage rate is 15-20% [1,2].

Most miscarriages occur during the first 7 weeks of pregnancy. Aneuploidy and unbalanced chromosomal abnormalities account for 50-60% of fetal loss during this period [3-6]. Cytogenetic evaluation of the products of conception (POC) is central in determining the cause of pregnancy loss and aids in the estimation of recurrence risk and in counseling for subsequent pregnancies. However, there are many challenges in cytogenetic evaluation. Recent studies have demonstrated that the type of tissue received by a cytogenetics laboratory is critical for the success of cell growth in culture and the subsequent karyotype analysis [5,7]. Our observations and those of others [5,7] demonstrate that the average culture success rate varies by tissue type with placental villi being the highest (>80%) and fetal parts being the lowest (<40%). Placental decidua almost always represents maternal tissue and is thus not an appropriate specimen type for study [7-10].

As generally perceived, the conventional chromosome analysis method is limited to obtaining results of numerical abnormalities and gross structural rearrangements. In contrast, CMA has a much higher resolution, detecting submicroscopic rearrangements as small as 50 kb by examining extracted DNA from the uncultured cells of fresh POC specimens [8,11-18]. A recent report entitled “rescue karyotyping” using chromosomal microarray analysis (CMA) on the DNA extracted from archived paraffin-embedded tissue after a procedure of dilation & curettage (D&C), has further proven that the DNA-based array method is effective enough to obtain critical fetal cytogenetic information from a prior loss, even if the loss occurred years earlier, in an assessment of couples with recurrent pregnancy loss [19]. In this study, we evaluated culture success rates in various types of POC specimens and the frequencies of chromosomal abnormalities by G-banding analysis in 5457 consecutive POC samples. We also performed an Oligo-SNP chromosomal microarray analysis (CMA) on 268 clinical cases in an attempt to compare test success rates and abnormality rates between the two methods.

## Methods

### Detection of chromosome abnormalities by the G-banding method

**Figure 1:**
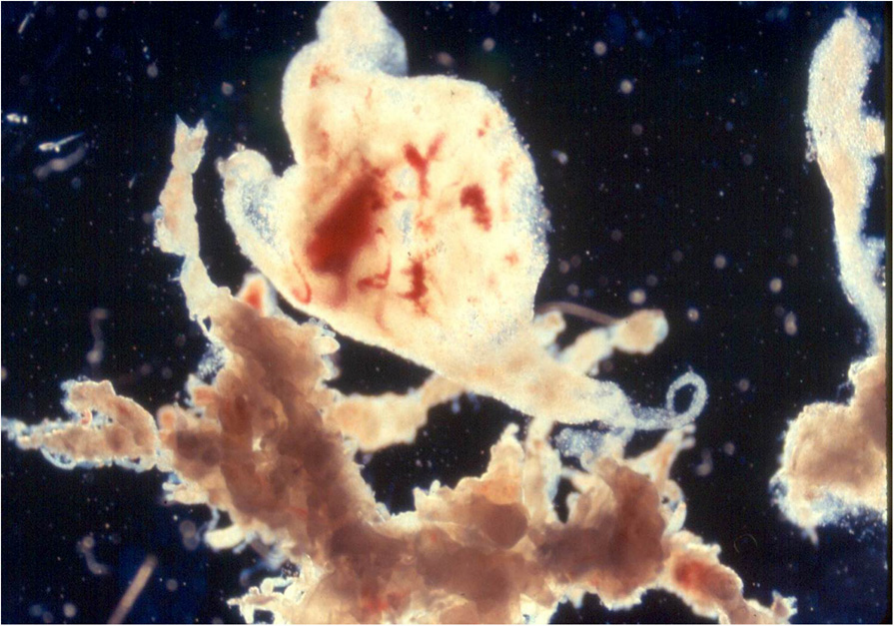
**Placental villi before cleaning (400x magnification).** Please note that there is a piece of maternal deciduous tissue attached to the villi (at the upper center).

**Figure 2:**
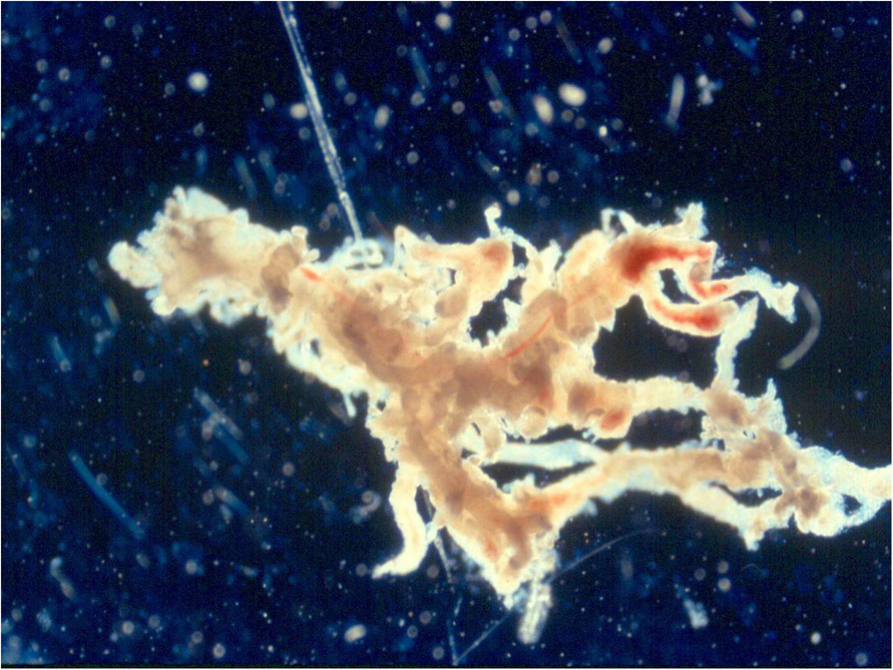
Placental villi after cleaning (400x magnification).

When received, the POC specimens were first placed under a dissecting microscope for gross examination. Cases with specimens that contained only placental decidua were excluded from the study since placental decidua is most likely maternal in origin. The specimens were then carefully dissected, and rinsed 3x in culture medium to remove maternal deciduous tissue before set-up for culture (Figures 1 and 2). Because of our extensive experience in processing chorionic villi samples (CVS) [20], we expect the risk for maternal cell contamination (MCC) to be minimal for specimens so processed. A total of 5457 consecutive POC specimens were then categorized as placental villi, fetal parts, and unspecified POC tissue (e.g., chorionic membrane, umbilical cord), cultured, harvested and analyzed by conventional cytogenetic analysis (the G-banding) method as described elsewhere. For specimens containing placental villi and other types of tissue, only placental villi were used for culture.

### Detection of genomic aberrations by the oligo-SNP chromosomal microarray

Each of the POC specimens was carefully examined, dissected and cleaned in the same way as mentioned above to remove maternal deciduous cells to reduce the risk of maternal cell contamination. Cases with specimens that contained only placental decidua were excluded from the study. A total of 268 clinical cases were analyzed by Oligo-SNP CMA (oligonucleotide, single nucleotide polymorphism, Affymetrix™ CytoScan HD, Affymetrix™, Inc., Santa Clara, California, USA). Among the cases studied, 32 cases were performed by both G-banding and array methods; 107 cases without a cytogenetic result due to failed culture were studied thereafter by the CMA method. The remaining 129 cases were performed by the array method alone. This Oligo-SNP CMA method used a microarray containing over 2.67 million probes, including 1.9 million copy number probes and 750 thousand SNP probes. The overall average inter-probe distance is 1,150 base pairs. Thresholds for genome-wide screening are set at >200 kb for gains, >50 kb for losses, and >10 Mb for regions of homozygosity (ROH).

## Results

**Figure 3:**
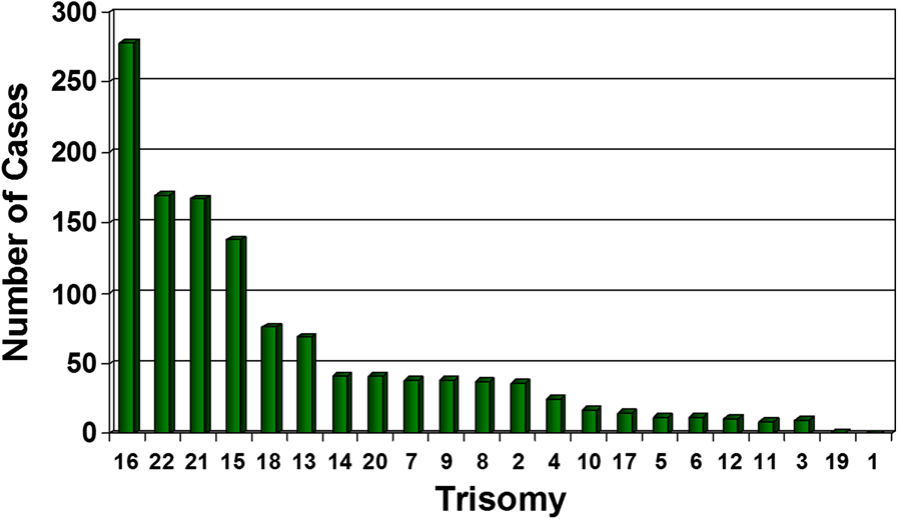
Autosomal trisomies (n = 1241) breakdown by chromosome.

The overall culture success rate for POC tissues was 75% (4092/5457), lowest for fetal parts (382/995; 38.4%) and highest for placental villi (2907/3567; 81%). The abnormality rate was 58% for placental villi, but only 25% for fetal parts. Of the abnormalities detected (N = 1872), the most common were aneuploidies including trisomy 16 (14.7%), triploidy (14%), monosomy X (13%), trisomy 22 (8.9%), trisomy 21 (8.6%), and trisomy 15 (7.3%), while the least encountered aneuploidies were trisomy 1, trisomy 19 and autosomal monosomies (except monosomy 21) (Table 1 and Figure 3).

**Table 1:** Frequency of chromosome abnormalities in POC with abnormal karyotypes (n = 1872)

**Type**	**Approximate proportion of abnormal karyotypes**
**Aneuploidy**	
Autosomal trisomy	1236 (66%)
Autosomal monosomy	19 (1%)
45, X	243 (13%)
**Triploidy**	262 (14%)
**Tetraploidy**	75 (4%)
**Other (inversions, translocations, etc.)**	37 (2%)

**Figure 4:**
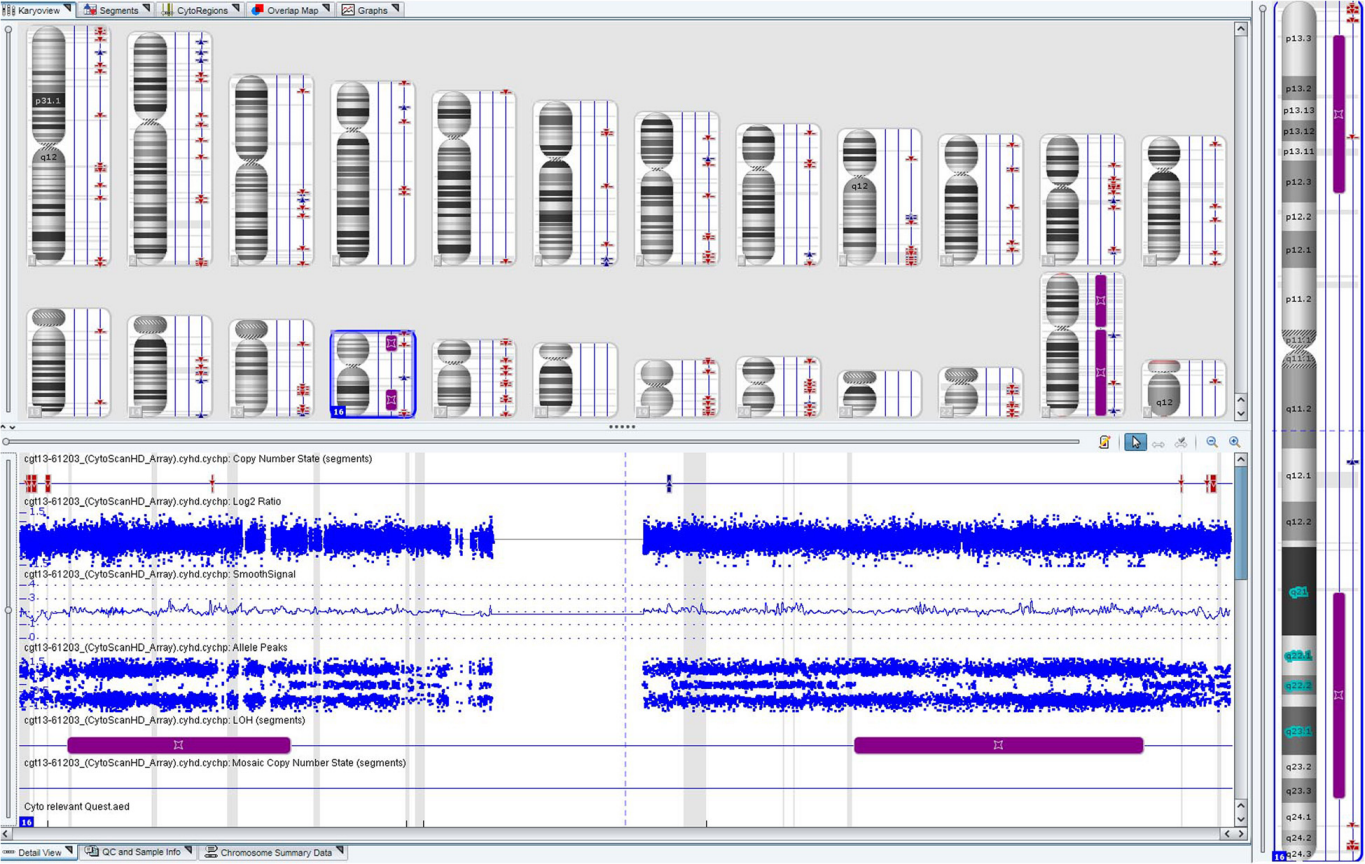
A case with segmental UPD 16 was detected by Oligo-SNP CMA.

**Figure 5:**
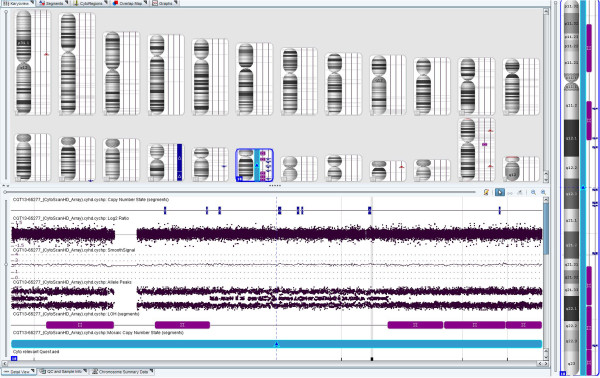
A case with a Xp21.1 deletion, trisomy 16 and mosaic segmental UPD 18 was detected by Oligo-SNP CMA.

In the Oligo-SNP CMA study series, the 32 cases analyzed by both G-banding and the array method demonstrated 81% (26/32) concordance, confirming all unbalanced abnormalities and detecting additional abnormalities in 5 cases not identified by the G-banding method, which included one case with a cryptic unbalanced (1;10) translocation (note: parental studies have been requested to determine its origin) and 4 cases with a VOUS result (variant of unknown significance). However, there was also one case that was reported as normal by the array method, but actually had a balanced (14;15) translocation (Table 2). Among the 107 cases with failed culture, this array method was successful in 84% (90/107) of cases and yielded a 39% (35/90) abnormality rate. In addition, one case with no copy number change was found to have segmental UPD 16 (41.7 Mb) (Table 2 & Figure 4). In the remaining 129 cases studied by Oligo-SNP CMA alone, testing was successful in 91% (118/129) of cases, yielded a 51% (60/118) abnormality rate and detected one case involving mosaic segmental UPD 18 (46.6 Mb) (Table 2 & Figure 5). Therefore, overall, POC specimens studied by CMA were successful in 89.6% (240/268) of cases and yielded a 44.6% (107/240) abnormality rate.

**Table 2:** The Oligo-SNP CMA results obtained from the 268 clinical cases

		**Microarray**				
		**Normal**	**Abnormal**	VOUS*	**Failed**	**Total**
**Karyotyping (chromosomes)**	**Normal**	19	1(a)	4(b)	0	24
	**Abnormal**	1(c)	7	0	0	8
	**No growth**	55	30(d)	5	17	107
	**CMA alone**	58	49(e)	11	11	129
	**Total**	133	87	20	28	268

(a) arr[hg19] 1p36.33p36.21(849,466-15,970,926)x1, 10q26.2q26.3(129,968,527-135,427,143)x3.

(b) arr[hg19] 1q21.1(145,368,364-145,829,474)x1, 2p16.3(50,909,653-50,971,464)x1.

arr[hg19]2q37.1(233,859,442-234,137,615)x3, 3q28q29(191,860,743-92,468,987)x1.

arr[hg19]Xq28(154,120,734-154,565,718)x2 (male).

arr[hg19]1p32.3(55,085,169-55,338,679)x3.

(c) 45,XX,der(14;15)(q10;q10).

(d) One case with segmental UPD 16

[arr[hg19] 16p13.3p12.3(89,560-20,228,889)*x*2 hmz,

16q21q23.3(62,222,293-83,741,752)*x*2 hmz].

(e) One case with a Xp21.1 deletion, trisomy 16, and mosaic segmental UPD 18.

[ arr[hg19] Xp21.1(31,746,498-31,987,991)x1,(16)x3,(18)x2-3,

18p11.31q12.1(5,218,469-29,237,765)hmz, 18q21.31q23(55,397,870-78,014,582)hmz].

*VOUS – Variant of unknown significance.

## Discussion

The distribution of chromosome aneuploidies, particularly the trisomies, observed in this study was similar to those of previous studies [5,21]. The least encountered aneuploidies were trisomy 1, trisomy 19 and monosomies (except monosomy 21). Non-mosaic trisomy 1 appears to be incompatible with even rudimentary fetal development and correlates with a diagnosis of blighted ovum [22-24]. There was only one case of trisomy 19 documented in our study series; there were no cases of trisomy 19 reported in previous first trimester studies of abortuses [25]. We suspect that trisomy 19, like trisomy 1, is embryonic lethal due to its high gene content [*http://www.ensembl.org, v36*]. With the exception of monosomy 21, we rarely observed autosomal monosomy in our study. We hypothesized that this may reflect the high lethality of gene insufficiency in early fetal development [1,2].

Placental villi yielded higher rates of culture success and a higher percentage of abnormal karyotypes than did other specimen types. However, a very small fraction of these abnormal karyotypes may be due to confined placental mosaicism (CPM) [20]. Our findings further support the request for placental villi, whenever possible, for POC chromosome studies. The higher abnormality rate in placental villi may have been the result of a higher percentage of viable abnormal cells available for culture. The lower abnormality rate in fetal parts may be due to maceration of abnormal fetuses before the time of miscarriage, causing cells to be less likely to grow in culture. We hypothesized that fetal parts from a chromosomally normal fetus tend to have a better chance to grow in culture and yield a chromosome result. As villi have the highest culture success rate, clinicians should be encouraged to submit placental villi whenever possible [5,7].

Our present study demonstrates that the DNA-based microarray technologies overcome many of the limitations of conventional cytogenetic analysis on POC specimens and enhance the test success rate (89.6% vs. 75%), the turnaround time (8 days vs.14 days) and the detection of submicroscopic chromosomal aberrations. Furthermore, the CMA approach uses extracted DNA, instead of cultured cells, which eliminates the considerable amount of time required for cell culture. CMA is not capable of detecting balanced rearrangements such as translocations, inversions and insertions; however, these types of abnormalities are unlikely to be related to the cause of a miscarriage. Overall, the oligo-SNP CMA method possesses a higher resolution in detecting unbalanced genomic aberrations than the conventional chromosome analysis. In addition, SNP-based microarrays can detect polyploidy (e.g., triploidy) and uniparental isodisomy (UPD) [26-28].

With the advent of the more sensitive DNA-based CMA technologies, the conventional cytogenetic analysis might be replaced by the new quantitative, microarray-based methods in the near future. However, as with any new technology, the microarray methods need to be carefully validated. The relatively expensive and sophisticated technical requirements may slow down the general adoption of the CMA [16,18,29,30]. In their recent studies, Baxter et al. (2013) proposed an integrated strategy to perform genetic analysis on POC specimens by a FISH (fluorescence in situ hybridization) with reflex array approach [31]. In this proposal, the authors advocated FISH analysis to detect the common abnormalities such as aneuploidy of chromosomes 13, 18, 21, X or Y with a reflex to CMA when FISH was normal. Of 100 abnormal cases they studied, they found that 46% were detectable by a FISH aneuploidy panel alone, leaving only 54% of the abnormal cases to be further studied by the relatively expensive microarray method. However, our FISH experience did not support such a strategy. In our experience, the FISH test failure rate could be as high as 28%. It was noted that the slides prepared for FISH from the macerated POC specimens were mostly poor in quality, thus leading to an even higher test failure rate than the conventional G-banding method (28% vs. 25%). Furthermore, this FISH with reflex array approach can be as costly as the CMA method, because many FISH probes have to be used in the study.

## Conclusion

Placental villi yielded a higher rate of culture success and a higher percentage of abnormal karyotypes than did other specimen types. Specimens containing only placental decidua most likely represent maternal deciduous tissue and are not recommended for POC studies. We believe that CMA is a viable alternative to the conventional G-banding method, even though CMA is relatively costly. Significant benefits of CMA, such as detection of submicroscopic genomic aberrations, elimination of time required for cell culture, a shorter turnaround time, a lower test failure rate and the robust nature of CMA, have been well demonstrated. In addition, CMA can be also performed to obtain a cytogenetic result on archived paraffin-embedded POC tissues [19]. At present, we routinely offer the Oligo-SNP CMA method to clients whenever there is a culture failure and a back-up specimen is still available. In the future, as the cost of CMA decreases, CMA testing may be the first choice for cytogenomic evaluation of POC specimens.

## Ethical approval and consent

These studies were performed on anonymized samples received in the clinical laboratory and thus were exempted from the requirement for consent by an opinion of the Western Institutional Review Board.

## Competing interests

The authors declare that they have no competing interests.

## Authors’ contributions

BTW designed and conducted the POC research project, drafted and finalized the manuscript. TC supervised the staff of the POC laboratory throughout the study period. FZB provided input to the POC project and made critical comments on the drafted manuscript. KAK/LPR gathered clinical information, compiled and analyzed the array data. MOH/MME/MHH/TS/JW reviewed cases referred for chromosome analysis and made critical comments on the drafted manuscript. RO initiated and carried out the first phase of the array studies. AA supervised the POC research project from its beginning, reviewed and interpreted the array results of most POC cases; made critical comments on the drafted manuscript. All authors read and approved the final manuscript.

## Authors’ information

Boris T. Wang first author.
